# DIFFERENCES IN PHYSICAL ACTIVITY AMOUNT AND FRAGMENTATION OVER 24 HOURS BY B-AMYLOID STATUS AND AGE

**DOI:** 10.1093/geroni/igac059.1306

**Published:** 2022-12-20

**Authors:** Francesca Marino, Fangyu Liu, Eleanor Simonsick, Qu Tian, Jennifer Deal, Amal Wanigatunga, Susan Resnick, Jennifer Schrack

**Affiliations:** Johns Hopkins Bloomberg School of Public Health, Washington, District of Columbia, United States; Johns Hopkins Bloomberg School of Public Health, Baltimore, Maryland, United States; National Institute on Aging, Baltimore, Maryland, United States; National Institutes on Aging, Baltimore, Maryland, United States; Johns Hopkins University, Baltimore, Maryland, United States; Johns Hopkins Bloomberg School of Public Health, Baltimore, Maryland, United States; National Institute on Aging, Baltimore, Maryland, United States; Johns Hopkins Bloomberg School of Public Health, Baltimore, Maryland, United States

## Abstract

β-amyloid is a hallmark of Alzheimer’s disease and can be measured in vivo by PET imaging. Whether PiB+ (elevated β-amyloid) individuals exhibit distinct physical activity patterns throughout the day (i.e., activity amount or fragmentation) is unknown. Using data from 125 BLSA participants (56-95 years) with PET imaging and ≥3 days of wrist accelerometry, we examined associations between PiB status and activity patterns daily and during 6-hour time blocks using multivariable linear regression models. Modification by age was also examined. Daily total activity counts and activity fragmentation did not differ by PiB status (p=0.34 and p=0.70, respectively). Yet, per each additional year of age, PiB+ individuals were more active during early morning (p=0.04) and evening (p=0.029) but trended towards less active in mid-morning (p=0.069) than PiB- individuals. Though replication of findings is needed, older PiB+ participants appeared more active in the late evening/early morning, possibly reflecting altered sleep-time, agitation, or restlessness.

